# EEG Microstates in Altered States of Consciousness

**DOI:** 10.3389/fpsyg.2022.856697

**Published:** 2022-04-27

**Authors:** Lucie Bréchet, Christoph M. Michel

**Affiliations:** ^1^Functional Brain Mapping Laboratory, Department of Fundamental Neuroscience, University of Geneva, Geneva, Switzerland; ^2^Department of Neurology, Harvard Medical School, Boston, MA, United States; ^3^Center for Biomedical Imaging (CIBM), Lausanne, Switzerland

**Keywords:** altered states of consciousness, EEG microstates, meditation, dreaming, mind-wandering, anesthesia

## Abstract

Conscious experiences unify distinct phenomenological experiences that seem to be continuously evolving. Yet, empirical evidence shows that conscious mental activity is discontinuous and can be parsed into a series of states of thoughts that manifest as discrete spatiotemporal patterns of global neuronal activity lasting for fractions of seconds. EEG measures the brain’s electrical activity with high temporal resolution on the scale of milliseconds and, therefore, might be used to investigate the fast spatiotemporal structure of conscious mental states. Such analyses revealed that the global scalp electric fields during spontaneous mental activity are parceled into blocks of stable topographies that last around 60–120 ms, the so-called EEG microstates. These brain states may be representing the basic building blocks of consciousness, the “atoms of thought.” Altered states of consciousness, such as sleep, anesthesia, meditation, or psychiatric diseases, influence the spatiotemporal dynamics of microstates. In this brief perspective, we suggest that it is possible to examine the underlying characteristics of self-consciousness using this EEG microstates approach. Specifically, we will summarize recent results on EEG microstate alterations in mind-wandering, meditation, sleep and anesthesia, and discuss the functional significance of microstates in altered states of consciousness.

## Introduction

Consciousness represents all that we subjectively experience, i.e., the qualitative feeling within an experience, e.g., the warmth of home or cherished memories of loved ones. An essential aspect of consciousness is its link with a self, which is the subject of conscious experience. The brain generates experiences day after day. Understanding the neuronal architecture that forms conscious experiences is one of the fundamental questions of cognitive neuroscience. More than 130 years ago, James (1890) pointed out that *“As we take a general view on the wonderful stream of our consciousness, what strikes us first is this different pace of its parts. Like a bird’s life, it seems to be made of an alternation of flights and perchings.”* Even though self-consciousness seems to be continuous as a stream of water, it appears to be composed of *“perchings,”* which may represent brain states with specific mental contents, and *“brief flights,”* which may be the fast transitions between mental states. Interestingly, a number of theories propose that consciousness may be parceled into discrete states lasting around 100–200 ms (Efron, 1970; Bressler, 1995; Rabinovich et al., 2001; Deco et al., 2011). Therefore, it seems that dynamics that underlie these subjective experiences have to describe the brain functioning in this short time range.

EEG measures the brain’s electrical activity with high temporal resolution (i.e., on the scale of milliseconds) and, therefore, might be used to investigate the temporal dynamics of conscious mental states (Michel and Koenig, 2018). Interestingly, Lehmann (1987) suggested that these short-lasting mental states may be represented by stable global patterns of electrophysiological brain activity, the so-called *“microstates of cognition”* or *“atoms of thoughts.”* The time window during which spatially distinct brain processes are accepted as a short EEG microstate lasts between 60 and 120 ms (Koenig et al., 2002; Michel and Koenig, 2018). While the most dominant EEG microstate maps are highly reproducible within and between participants (Koenig et al., 2002; Khanna et al., 2014; Tomescu et al., 2018; da Cruz et al., 2020; Zanesco et al., 2020), their temporal dynamics are sensitive to the momentary state of the brain. Altered states of consciousness (e.g., sleep, disorders of consciousness, anesthesia, hypnosis, or meditation) influence the temporal dynamics of microstates (Katayama et al., 2007; Brodbeck et al., 2012; Panda et al., 2016; Faber et al., 2017; Stefan et al., 2018; Shi et al., 2020; Bréchet et al., 2021). The inner self is unique to each person. We may assume that others have similar inner experiences, but so far, we cannot directly measure the inner self of others. In this perspective, we are going to argue that it is possible to explore the underlying characteristics of self-consciousness and summarize our current findings. Recent experimental studies have focused on establishing a link between subjective conscious experiences and measurable neuronal activity. We will question “How can we capture the self-relevant, conscious thoughts of the wandering mind?” then we will ask 2. “Can meditation alter conscious brain states?” 3. We will assess 3. “What happens with conscious experiences during sleep?” and finally 4. “How does consciousness fade-out during anesthesia?”

## The Concept of EEG Microstates

One way to globally represent the momentary brain activity resulting from concomitant active brain areas is to record EEG with a whole-scalp array of electrodes and map the potential scalp field at each moment in time (Lehmann, 1987; Michel et al., 2009). By inspecting the temporal evolution of these potential scalp maps of ongoing resting state EEG, Dietrich Lehmann made the seminal observation that the spatial configuration (the topography) of the potential map remains stable for short periods of time and then rapidly switches to a new configuration in which it remains stable again. He called these periods of stability, which lasted around 100 ms, the EEG microstates, where the term “micro” refers to the temporal (the briefness) and not to the spatial scale. In 1990, Lehmann suggested that these brief episodes of topographic stability of the global electric field represent the “atoms of thought” (Lehmann, 1990), without yet strong supporting evidence for this analog.

A commonly used approach to determine the most dominant topographies in spontaneous EEG is cluster analysis (Pascual-Marqui et al., 1995). When applying this data-driven method, a striking observation is that a few prototypical maps dominate the recorded signal, explaining around 70–80% of the variance (Koenig et al., 2002; Custo et al., 2017). While the topography of these maps is very similar across studies (for reviews see Khanna et al., 2015; Michel and Koenig, 2018), the most fundamental observation is that these few maps are not randomly appearing in time, but they remain stable for short periods of around 80–150 ms (Koenig et al., 2002). Thus, the ongoing spontaneous EEG is parceled into short segments represented by one specific topography that repeats in time in variable sequence and duration. Given the above described theories of parcellation of consciousness in discrete states that last around 100–200 ms, it is intriguing to assume that EEG microstates are the electrophysiological manifestation of these conscious states (Bressler and Kelso, 2001; Changeux and Michel, 2004), representing short periods of synchronized activity of large-scale functional networks (Seeber and Michel, 2021).

## EEG Microstates in Self-Related Mind-Wandering

The temporal structure of spontaneous mentation is key to forming a meaningful stream of consciousness. The human perception appears continuous, dynamic, and unsegmented (Zacks et al., 2001). Neuroscience research embraced the tremendous attention paid to the resting brain’s activity and dramatically changed our view on mind-wandering (Smallwood and Schooler, 2015; Christoff et al., 2016). Until now, the precise functional role of resting-state networks, which may represent distinct states of consciousness, remains unclear. Previous studies have tried to relate EEG microstates to ongoing mental activity. For example, Seitzman et al. (2017) altered the temporal features of the 4 canonical microstates by instructing their participants to either mentally subtract numbers or to spontaneously mind-wander. They found a significant decrease in occurrence (i.e., the frequency of occurrence independent of its individual duration) and duration (i.e., the average duration that a given microstate remains stable) of microstate C and an increase in microstate D during mental calculation, supporting the hypothesis that state D is related to the attentional system. In addition, Milz et al. (2016) showed increased coverage (i.e., the fraction of total recording time for which a given microstate is dominant) of microstate A while visualizing and coverage of microstate B while verbalizing. Thus, these studies show that the temporal dynamics of EEG microstates may be sensitive to instruction and changes in the content of spontaneous mentation.

A few studies have examined associations between microstates and spontaneous thought using a retrospective questionnaire administered to participants after a short period of rest. Pipinis et al. (2017) found a negative association between microstate C and experienced somatic awareness (SA) of the subsequent Amsterdam Resting-State Questionnaire (ARSQ). SA was evaluated by questions related to bodily self-consciousness (e.g., “I was conscious of my body”; “I thought about my heartbeat”; “I thought about my breathing.” In a follow-up study, Tarailis et al. (2021) showed that microstate F was associated with SA, microstates C, E, and G were related to the comfort domain and microstates B and C related to the self-domain. Zanesco et al. (2021b) directly examined the association between episodes of mind wandering and microstates in a cognitive task with embedding experience sampling probes to capture moments of on-task vs. off-task focus. The authors found that self-reported mind wandering and response time variability differentiated pre-stimulus EEG microstate dynamics during a sustained attention task. Further, Zanesco et al. (2021a) found associations between self-reported aspects of spontaneous thought and temporal parameters of EEG microstates and thus pointed out the relevance of using retrospective questionnaires for understanding intrinsic brain activity.

**FIGURE 1:**
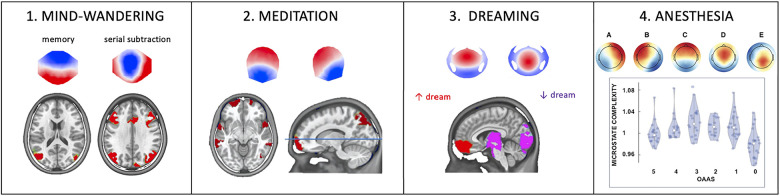
The influence of different functional brain states on EEG microstates. **(1)** Instructing subjects to focus their thoughts on an autobiographical memory increased duration and occurrence of one microstate, while focusing the attention on a serial subtraction task increased duration and occurrence of another microstate. Source localization attributed the memory-specific microstate to the lateral parietal lobe and the arithmetic-specific microstate to the activity of a frontoparietal network (Figure modified with permission from Bréchet et al., 2019). **(2)** Compared to placebo, six weeks of digital meditation training led to a reconfiguration of two of the four predominant microstates whose generators were found in the superior frontal gyrus, the superior temporal gyrus, the insula, the left inferior, and the right superior parietal lobule (Figure modified with permission from Bréchet et al., 2021). **(3)** EEG microstates during dreaming: compared to awake, Non-Rapid Eye Movement Sleep (NREM), dominated by slow-wave activity, selectively increased the Global Explained Variance and duration of two out of five microstates which were localized in the medial and middle frontal gyrus and the posterior cortex and midbrain, respectively. Interestingly, dreaming during NREM sleep further increased the presence of the frontal microstate but decreased the presence of the posterior microstate, indicating a local awakening (less slow-wave) of the posterior cortex (Figure modified with permission from Bréchet et al., 2020). **(4)** EEG microstate during global anesthesia. All five microstates identified in this study were modulated in the same way by different levels of Propofol: they first showed an increase in occurrence and complexity and decrease in duration with moderate sedation, but an inverted behavior with deeper sedation. This u-shaped behavior might be linked to the paradoxical excitation induced by moderate levels of Propofol. OAAS refers to the Observer’s Assessment of Alertness/Sedation scale ranging from 5 (awake) to 0 (deep anesthesia) (Figure modified with permission from Artoni et al., 2022).

Our recent study (Bréchet et al., 2019) examined the spatiotemporal dynamics of large-scale brain networks associated with particular thoughts. Participants were instructed to direct their thoughts to their past, self-related memories or mental, self-unrelated calculation to examine the large-scale networks underlying the internal conscious thoughts. We examined the spatiotemporal dynamics of brain activity with high-resolution 7-Tesla fMRI and high-density EEG in two separate sessions. We expected the fMRI data to confirm that the default mode network comprises distinct sub-systems related to visual imagery and autobiographical memory retrieval. We then hypothesized that the EEG microstate analysis would reveal that these functional sub-networks are not continuous but relatively temporally parsed. First, the fMRI data confirmed that sub-networks of the default mode network are activated during episodic memory retrieval, and these subnetworks show distinct connectivity patterns. Second, EEG microstate analysis showed two microstates that increased in duration and occurrence during autobiographic episodic memory retrieval and another microstate during mental arithmetics (Figure 1-1).

Interestingly, while the sources of the two microstates that dominated during memory retrieval were very similar to the two fMRI resting state networks, the temporal analysis revealed a continuous switching between the two networks over time in the sub-second range. The microstate analysis thus allowed to disentangle the sub-parts of thoughts related to the conscious experience of episodic autobiographic memory, i.e., visualization of the scene and the self. Using functional connectivity analysis in the source space, we showed in a follow-up study that autobiographical memory retrieval emerges during a precise theta-gamma phase-amplitude coupling between the medial temporal lobe and the prefrontal and the posterior cingulate cortex (Roehri et al., 2022).

Sometimes the conscious mind moves spontaneously from one thought to another. However, at other times, it keeps coming back to the same thought, drawn by a particular past event or an emotional experience. While a healthy person naturally balances between different types of thoughts that make sense to both the individual and the world, someone with a mental health issue may lose control over the natural flow of the wandering mind and continuously cycles through a series of rigid thoughts that are at odds with reality. To capture the stream of ongoing thoughts is challenging yet essential to better comprehend the composition of healthy and pathological thoughts. Several studies have shown that the temporal dynamics of EEG microstates are modulated by pathologies that lead to aberrant mind states such as schizophrenia or depression (Lehmann et al., 2005; Tomescu et al., 2015; Rieger et al., 2016; Damborska et al., 2019; da Cruz et al., 2020; Murphy et al., 2020).

## EEG Microstates in Self-Focused Meditation

Brain activity constantly fluctuates in and out of different mental states that are stable for fractions of seconds. Only one epoch or state of conscious content can be present at a time (Seth and Baars, 2005). If EEG microstates are indicative of the level of consciousness, then they can be modulated by different states of mind. Panda et al. (2016) recorded simultaneous EEG-fMRI while experienced participants meditated. This study showed that at rest, the meditators exhibited increased duration and occurrence of DMN related microstate C, which further increased during meditation. Katayama et al. (2007) showed that a decrease in microstate C characterized light hypnosis, while deep hypnosis was associated with a reduction of microstate D. The study of Faber et al. (2017) tested two phases of transcendental meditation—transcending (i.e., self-awareness becomes primary) and undirected (i.e., the mind becomes engaged in an undirected stream of thoughts) mentation—and compared them using EEG microstates. Compared to the transcending mentation, undirected mentation was marked by significantly higher coverage and occurrence of microstate C. In comparison, transcending meditation was characterized by higher coverage and occurrence of microstate D.

To examine the effects of internally self-focused meditation, we analyzed the resting-state 64-channel EEG of the participants reported in Ziegler et al. (2019). In this follow-up study (Bréchet et al., 2021), we used the EEG microstate approach to capture resting-state network dynamics before and after the meditation training and compared them to changes before and after placebo sessions. Compared to a placebo condition and the pre-meditation resting EEG data, distinct new microstate topographies appeared after 6 weeks of intensive meditation training. In addition, source analysis identified the fronto-insular-parietal network, including the right insula, superior temporal gyrus, parietal lobule, and frontal gyrus bilaterally (Figure 1-2). These brain areas are involved in self-related, multisensory conscious experiences. Our results thus indicate that EEG microstates can capture and monitor sustained changes in conscious mentation induced by breath-focused digital meditation practice and open new avenues for developing novel approaches to treat neuropathological states such as anxiety, hyperactivity, or depression.

## EEG Microstates in Sleep

Most of us dream every night, although we are unlikely to remember any of our dreams. When we sleep, our brains repeatedly cross a boundary between unconsciousness and dreaming—a particular form of consciousness. Why are we sometimes unconscious, while at other times, we have conscious experiences in the form of dreams? Which brain states determine whether dreams will occur and what prevents us from waking up during these conscious experiences? Clear evidence shows that dreaming can occur in both rapid eye movement (REM) and non-rapid eye movement (NREM) sleep (Stickgold et al., 2001; Nir and Tononi, 2010; Siclari et al., 2018). In a recent study (Bréchet et al., 2020), we showed that two specific microstates dominate during NREM sleep compared to awake, but that these two microstates are differently affected by dreaming during NREM sleep: While microstate D that includes generators in the occipital cortex, the thalamus, and the brainstem becomes activated, i.e., reduced presence of state D during dreaming (“the awakening of the posterior hot zone”), there is an increased deactivation, i.e., increased presence during dreaming of microstate C that encompasses prefrontal brain regions (Figure 1-3). The former may account for conscious experiences with rich perceptual content, while the latter may account for why the dreaming brain may undergo executive disconnection and remains asleep. This study thus suggests that NREM sleep consists of alternating brain states whose temporal dynamics determine whether conscious experience in terms of dreams arises.

## EEG Microstates Under Anesthesia

The model put forward in this perspective is that conscious experience relies on the brain’s ability to generate a stream of short-lasting global functional states characterized by the synchronized activity of large-scale networks. The EEG microstates are the electrophysiological correlates of these basic building blocks of conscious content. Notably, the temporal dynamics of these momentary states in terms of their frequency of occurrence, duration, and the syntax of transition between them have been demonstrated to be sensitive to changes in the content of conscious experiences (Michel and Koenig, 2018). The temporal dynamics of microstates are based on metastability that allows for a continuous balance between stability and disorder and a rapid and flexible switch from one state to the other (Tognoli and Kelso, 2014). Any change in the typical temporal structure of state transitions, whether prolonged staying in a given state or disordered transitions between states, will likely result in alterations in the global state of consciousness.

To directly test the modifications of the microstate’s temporal dynamics by altered states of consciousness, we investigated the spatio-temporal properties of EEG microstates in 23 surgical patients from their awake state to unconsciousness, induced by stepwise increasing concentrations of the intravenous anesthetic propofol (Artoni et al., 2022). Loss of consciousness was characterized by increasing duration and spatial correlation of all microstates (i.e., the mean spatial correlation of the microstate map with the global field power peak maps within the spatially filtered dataset), decreasing occurrence of all microstates, and reduced complexity assessed by the Lempel-Ziv complexity index (i.e., a more heterogeneous succession of microstates) of the microstate sequences. A similar EEG microstate complexity measure has been recently tested for aiding early diagnosis of Alzheimer’s disease (Tait et al., 2020). Interestingly, we found a U-shaped effect of propofol from baseline to light sedation and from sedation to surgical anesthesia. Light sedation was characterized by more diverse spatiotemporal EEG microstate patterns with shorter duration, higher density, lower spatial correlation of the microstates, and increased complexity (i.e., reflect simpler and repetitive microstate sequences) of the microstate sequences than awake and deep anesthesia (Figure 1-4). This peculiar behavior is probably linked to the paradoxical excitation effect of propofol and other anesthetics at a lower dose, marked by hyperexcitability, disinhibition, loss of effective control, and probably greater awareness of both inside and outside stimuli (Fulton and Mullen, 2000). Such an altered state of consciousness has also been described after using “magic mushroom”—psilocybin, i.e., a naturally occurring psychedelic prodrug, as the “entropic brain” state (Carhart-Harris et al., 2014).

## Discussion

The inner self is unique to each person, who is the only one who can access this state. An observer may distinguish various conscious states of others, but the *“self-experience of how it feels”* is restricted to the inner self of the involved person. As James (1890) described, “*when I wake up, I do not have to question ‘who am I,’ because I still have my own thoughts that seem to be continuous and represent my personal past.”* One may assume that others have similar inner experiences, but nobody else can directly experience the inner self of others. In this brief perspective, we have argued that it is possible to examine the underlying characteristics of self-consciousness using the EEG microstates approach. Specifically, we summarized recent EEG microstate alterations in mind-wandering, meditation, sleep, and anesthesia. We presented results from our recent study that showed that the microstate analysis allows us capture the conscious experience of episodic autobiographic memory. We then used the EEG microstate approach to capture resting-state network dynamics before and after the meditation training and showed distinct new microstate topographies after the intensive meditation training, which opens new possibilities for treating neuropathological states such as depression or anxiety.

In another study, we claim that the NREM sleep consists of alternating brain states whose temporal dynamics determine whether conscious experiences, i.e., dreams, may arise or not. Finally, we examined the temporal dynamics of EEG microstates from awake state to the loss of consciousness, which allowed us to capture an altered state of consciousness.

Consciousness is detectable in the behavior of others, but none of the neuroimaging techniques can thoroughly read the mind of private thoughts (Lee and Kuhl, 2016). While functional neuroimaging studies using fMRI demonstrated that the brain is intrinsically organized in large-scale networks, the relation between these slowly fluctuating networks and the cognitive activities is questioned because neuronal networks dynamically re-organize in the sub-second time-scale. EEG microstates show such sub-second re-organization and are thus better suited to study the dynamics of spontaneous conscious processes, such as what happens during mind-wandering, dreaming, meditation, or under anesthesia. A better understanding of the relation of EEG microstates to the content of conscious processes and their influence by the global mental state of the brain enables us to elucidate the sensitivity and specificity of these EEG signatures. Furthermore, since EEG is directly related to neuronal activity, a more profound analysis of the relation of EEG microstates to power in different frequency bands (Koenig et al., 2001; Férat et al., 2022) and mechanisms of communication between brain regions will give new insights into the neuronal mechanisms underlying the emergence of conscious thoughts and experiences, and will ultimately help to understand better what happens during altered states of consciousness and what goes wrong in the mind of those with a mental illness.

## Author Contributions

LB and CM wrote the manuscript. Both authors contributed to the article and approved the submitted version.

## Conflict of Interest

The authors declare that the research was conducted in the absence of any commercial or financial relationships that could be construed as a potential conflict of interest.

## Publisher’s Note

All claims expressed in this article are solely those of the authors and do not necessarily represent those of their affiliated organizations, or those of the publisher, the editors and the reviewers. Any product that may be evaluated in this article, or claim that may be made by its manufacturer, is not guaranteed or endorsed by the publisher.
